# 3D Oleophilic Sorbent Films Based on Recycled Low-Density Polyethylene

**DOI:** 10.3390/polym16010135

**Published:** 2023-12-31

**Authors:** Junaid Saleem, Zubair Khalid Baig Moghal, Gordon McKay

**Affiliations:** 1 Division of Sustainable Development, College of Science and Engineering, Hamad Bin Khalifa University, Qatar Foundation, Doha 34110, Qatar; gmckay@hbku.edu.qa; 2 Center for Advanced Materials, Qatar University, Doha 2713, Qatar; zubairkhalid009@gmail.com

**Keywords:** LDPE waste, oil spill, sorbent, 3D structure, plastic pollution, oil uptake

## Abstract

Recycling low-end, one-time-use plastics—such as low-density polyethylene (LDPE)—is of paramount importance to combat plastic pollution and promote sustainability in the modern green economy. This study valorizes LDPE waste by transforming it into 3D oleophilic swellable thin films through a process involving dissolution, phase separation, and extraction. These films are subsequently layered using a customized polypropylene (PP) based nonwoven fabric separator and securely sealed in a zigzag pattern. The zigzag-shaped seal enhances the adhesion of pollutants to the sorbent by providing wire curvatures that increase retention time and uptake capacity. As a result, the sorbent exhibits impressive oil uptake capacities, with immediate and equilibrium values of 120 *g*/*g* and 85 *g*/*g*, respectively. Notably, the as-prepared sorbent demonstrates low water retention and high selectivity for oil, outperforming commercially available oil sorbents. The unique design involving a 3D-film structure, superposed films, and a zigzag-shaped seal offers a sustainable and value-added solution to the issues of LDPE waste and oil spills on water surfaces.

## 1. Introduction

Plastics, enabled by large-scale production in the last century, have transformed the world [1,2]. However, they also pose a significant environmental threat due to their non-biodegradable nature and the challenges associated with recycling and reuse [3,4,5,6,7]. Additionally, the wide variety of plastic grades and high energy requirements make plastic reprocessing and recycling more complex compared to metals or glass. This has led to issues like seabirds ingesting plastic waste in the oceans, contributing to the problem of white pollution [8,9].

Among various plastic types, polyolefins, such as polypropylene (PP) and polyethylene (PE), are the most widely used, making up almost half of the global plastic production [10,11,12]. However, only a small fraction (9–10%) of plastics are recycled [13,14]. A 2017 report underscores that plastic packaging materials lose 95% of their value after a single use, resulting in an annual economic loss of USD 120 billion [15]. Specifically, the global thermoplastic polyethylene market reached approximately 110.13 million metric tons in 2022, with projections indicating an increase to roughly 135.08 million metric tons by 2030 [10]. Consequently, there is significant commercial potential, particularly in the field of oil sorption, when considering these materials.

Oil sorption has gained attention among various oil removal techniques, including physical, chemical, and biological methods [16,17,18]. Different adsorbents have been employed, but many face challenges like low oil selectivity, environmental concerns, and low sorption efficiency. To address these limitations, there has been a growing interest in designing customized oil sorbents [16,19]. Various materials, including natural fibers, synthetic polymers, and other substances, have been utilized as oil sorbents [19,20,21,22]. However, many of these have limitations in terms of binding capacity, oil retention, and buoyancy [23,24]. On the other hand, highly effective 3D carbon frameworks, such as carbon foams and aerogels, have proven efficient but are hindered by costly production processes [3,25].

Natural fibers have primarily been used as loose fibrous oil sorbents [26,27]. The main shortfall of loose fibrous assemblies is the inability to easily separate the fibers from the spill after use. The development of non-woven sorbents could address this problem [20,28,29,30,31]. Researchers developed oil sorbents by grafting polystyrene (PS) onto polyurethane foams, polypropylene (PP) and polyethylene (PE) powders, and polyurethane (PU) foams [32,33]. PET aerogel oil sorbent [34] has been developed from plastic waste with limited oil absorption capacity. Macroporous and microporous reusable oil sorbent films have been made from waste PE and PP [3,12,35,36,37,38].

There have been efforts to repurpose plastics, particularly LDPE waste, for oil–water separation and oil sorption applications. Superhydrophobic membranes from PP and LDPE wastes were fabricated using a two-step technique that utilizes bio-based solvents for dissolution and thermally induced phase separation (TIPS) for effective oil–water separation [39]. Microfiltration membrane was fabricated from acrylic fiber (AF) and LDPE shrink film wastes using an electrospinning technique [40]. Hydrophobic aerogels from LDPE were synthesized via vacuum drying [41]. A gravity-based separation sheet was made using reduced graphene oxide and LDPE through hot-pressing for oil–water separation [42]. Hydrophobic and oleophilic amine-functionalized graphene and LDPE nanocomposite were prepared for oil–water separation [43]. An LDPE hydrophobic monolith was made via the TIPS method, followed by vacuum drying for oil–water emulsions [44]. A hydrophobic and oleophilic mesh that separates oil from water continuously in situ via capillary action using LDPE was synthesized for oil–water separation [42]. It is evident from these examples that LDPE waste is a viable feedstock for oil sorption.

We have previously repurposed high-density polyethylene (HDPE) waste into oleophilic porous thin films using the extrusion–stretching–extraction route [12,35,36,37,38]. In this study, we valorize LDPE waste by transforming it into 3D oleophilic swellable thin films, which are subsequently layered using a customized nonwoven fabric separator and securely sealed in a zigzag pattern. The zigzag-shaped seal enhances the adhesion of pollutants to the sorbent by providing wire curvatures. The distinctive design optimizes the sorbent’s ability to efficiently absorb and retain oil, making it particularly useful for the removal of a thin layer of oil from water surfaces. Moreover, given that LDPE films tend to fold and shrink when they come into contact with oil–water emulsions, the introduction of nonwoven fabric support ensures that the sorbent maintains its structure. Furthermore, we conducted a comparison with commercially available oil sorbents to demonstrate the effectiveness of the as-prepared sorbent.

## 2. Experimental Section

### 2.1. Materials

Low-density polyethylene plastic waste (LDPE), including items such as single-use disposable polybags, was purchased from local market and used as LDPE. The nonwoven polypropylene fabric was purchased from the local market. An isomeric mixture of xylene (99.0% pure) was purchased from VWR Chemicals (US) and used without further purification. Sodium chloride (NaCl) was used as a filler and sieved to a size of 150–200 μm. Glass substrate plates were cut to 5 cm × 5 cm and mounted on the chuck of the spin coater. 

### 2.2. Methods

Spin-casting was performed using an Ossila spin coater, and the customized chuck was obtained from Ossila Instruments, Sheffield, UK. Heating and annealing were carried out using a MINO/30/TDIG hot air oven from Genlab Ltd.(Cheshire, UK). and a Heidolph magnetic hotplate stirrer (Schwabach, Germany). Scanning electron microscope (SEM) images were obtained with an FEI Quanta650FEG (Oregon, Hillsboro, OR, USA). FTIR analysis was conducted using the PerkinElmer TGA 8000™ and STA systems coupled instrument (PerkinElmer, Waltham, MA, USA). Atomic force microscopy (AFM) analysis was carried out using an AFM Atomic force microscope MFP-3D system (Asylum Research, Santa Barbara, CA, USA) equipped with a cantilever = -containing sharp tip (Al reflex coated Veeco model-OLTESPA, Olympus, Japan) can be used for obtaining detailed information about 3D surface topography of scanning areas in the range 0.5 µm^2^–90 µm^2^ with maximal resolution of 4096 points ∓ lines. Optical contact angles were calculated using OCA 35 from Dataphysics Instruments GmbH in Filderstadt, Germany. Tensile strength was measured using a friction/peel tester from Lloyd Instruments Ltd., Bognor Regis, UK. Thickness was measured in micrometers and was and cross-checked using a Deflesko FS3 PosiTector 6000 with a ferrous metal base.

### 2.3. Sorbent Preparation

LDPE plastic waste was washed with soapy water to remove dirt or oil; 1.3 g of LDPE was placed in a conical flask and dissolved in 10 mL of xylene at 110 °C for 20 min, or until a clear solution was achieved. Then, 2.6 g of sodium chloride with a particle size of 150–200 µm was added to the polymer solution and stirred for 10–15 min or until a homogeneously dispersed solution was achieved. Simultaneously, a 5 cm × 5 cm glass plate was heated to 100 °C and placed on the chuck of the spin coater.

The hot solution was poured onto the hot glass plate and allowed to spin. The spin-casting was programmed in three steps to achieve a uniform thin film: (a) a rotational speed of 300 rpm for 10 s, (b) a rotational speed of 900 rpm for 60 s, and (c) a rotational speed of 2800 rpm for 60 s. Excess polymer and solution were collected through a drain. The glass substrate with the thin film was removed from the chuck and annealed at 105 °C in a hot air oven for 20 min. The swellable thin film was then separated from the glass substrate and washed with water to remove the filler.

To prevent solvent exposure to the environment, the stirrer and spin coater were placed side by side in the fume hood. Three stages were programmed for the spin-casting. Due to the ease of binding the polymer to the glass substrate, the speed was kept low in the first stage. The polymer spread uniformly at medium speed following the next stage. The speed was further increased in the last step to remove the solvent molecules from the thin film and refine the thickness.

The concentration of filler can be altered with respect to the amount of polymer used. For example, 1 g of filler in 10 mL is considered 100 mg/mL, and the other concentrations were adjusted accordingly. Similarly, a specific sieve was used to determine the size of the filler.

The optimum conditions were selected based on the above experiments, and they are as follows: polymer 130 mg/mL, filler 260 mg/mL, size of filler 150–200 microns, third-round speed during spin-casting 2800 rpm. All the reported results, including SEM, AFM, and oil sorption studies, were obtained under these conditions.

**Figure 1:**
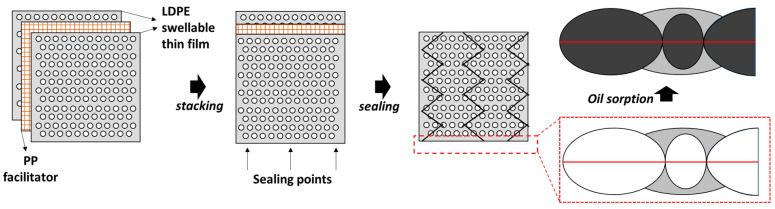
Zigzag sealing of LDPE swellable thin films and nonwoven PP sheets.

**Figure 2:**
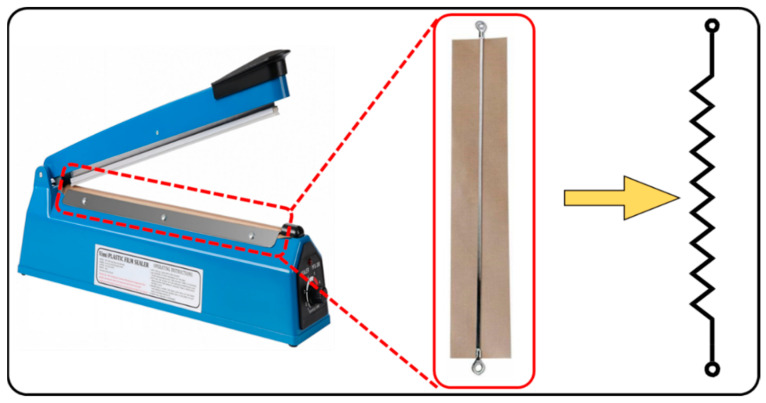
Zigzag-shaped sealant preparation.

A nonwoven polypropylene (PP) fibrous sheet measuring 5 cm × 5 cm underwent a softening process in a hot air oven at 80 °C for 5 min. This treatment was aimed at enhancing the sheet’s flexibility and compatibility with the low-density polyethylene (LDPE) swellable thin film, which was constructed from recycled plastic waste. Subsequently, the softened nonwoven PP fibrous sheet was strategically inserted between the swellable thin films. Specifically, the first sorbent thin film was laid down, followed by the placement of the nonwoven fabric, and finally, the second sorbent thin film was added (see Figure 1). These layers were then securely sealed in a zigzag pattern, as depicted in Figure 2. A common heat-sealing machine was purchased from a local market. A copper wire with a thickness of 0.8 mm was bent at 1 cm intervals to create a zigzag-shaped wire. The straight copper filament or wire from the machine was removed and replaced with the zigzag-shaped copper wire. The remaining fittings were carried out as before.

Initially, we investigated the oil separation studies using thin film sorbents made from 1:2 ratio of polymer to sodium chloride. We characterized LDPE sorbent using scanning electron microscopy (SEM), Fourier-transform infrared spectroscopy (FTIR), X-ray diffraction (XRD), and differential scanning calorimetry (DSC).

## 3. Results and Discussion

LDPE demonstrates lower strength compared to high-density polyethylene (HDPE) and PP. It struggles to maintain structural integrity as effectively as its counterparts, often folding and wrapping easily, which reduces the surface area. This issue becomes more pronounced when LDPE films come into contact with oil–water emulsions. To address this challenge, nonwoven PP fabric was introduced to provide structural support, enhancing the sorbent’s contact with the oil. The nonwoven fabric was strategically positioned between the prepared LDPE films and securely sealed in a zigzag pattern. This unique sealing method not only strengthened the adhesion of pollutants to the sorbent by introducing wire curvatures but also optimized the sorbent’s efficiency in absorbing and retaining oil. This design proved highly effective in removing thin oil layers from water surfaces.

**Figure 3:**
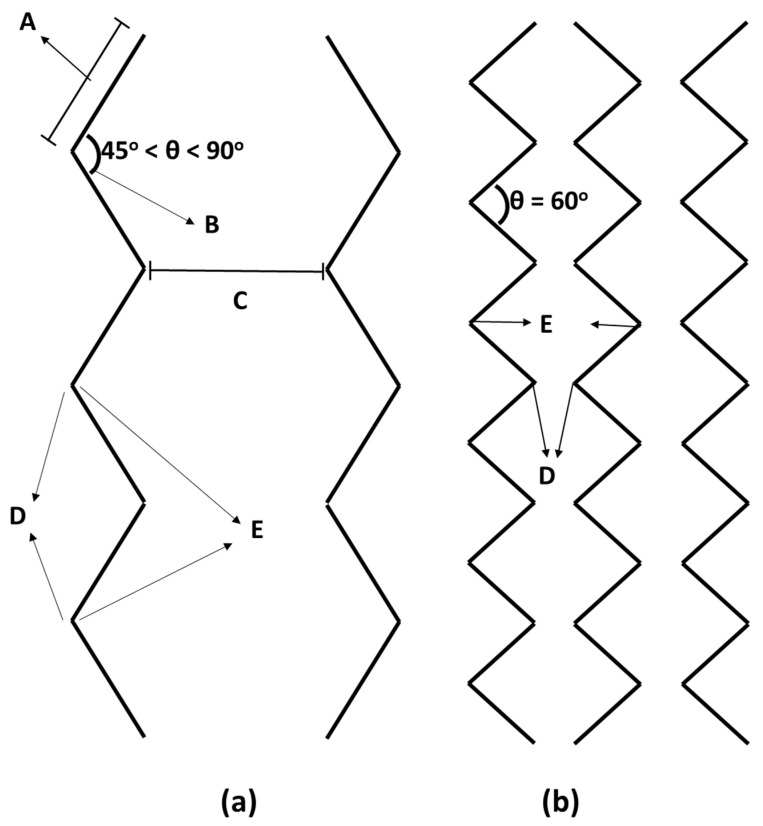
Illustration of zigzag sealing (**a**) showing the parameters in the arm, and (**b**) showing the parameters between the two arms.

Initially, we experimented with straight-line seals, leading to faster oil uptake and removal but proving inefficient for long-term oil retention. Consequently, we explored a zigzag sealing pattern to enhance both retention time and sorption capacity. The zigzag-shaped heat seal is illustrated in Figure 3. It consists of sealing arm portions and opposite receiving arm positions, and each arm portion is defined by interlocking peak tips and valley foots. The zigzag-shaped heat seal has peak tips of adjacent seals facing each other to improve retention. Arm Portion (A) is the distance between one curvature and the other curvature of the same seal, and Angle (B) represents the geometry or space between two intersections of two arm portions at or near the point where they meet. This angle is 60° but should ideally be kept between 45° and 90°. A seal is a region where two or more films are connected by heat, and the distance between two adjacent seals is labeled as C. Peak Tip (D) is the outer surface of the bending caused by zigzag sealing, while Valley Foot (E) is the inner surface of the bending created by zigzag sealing. Both the length of the arm portion and the distance between two peak tips created by the zigzag-shaped heat seal are 5 mm for a 1600 mm^2^ area, with a heat seal diameter of 1 mm or less. 

The parameters of the zigzag-shaped heat seal that significantly impact pollutant retention are as follows:

- Angle between two arms of the zigzag-shaped seal.
- Length of the arm in the zigzag-shaped seal.
- Diameter of the arm in the zigzag-shaped seal.
- Distance between two opposite arms of the zigzag-shaped seal.

**Figure 4:**
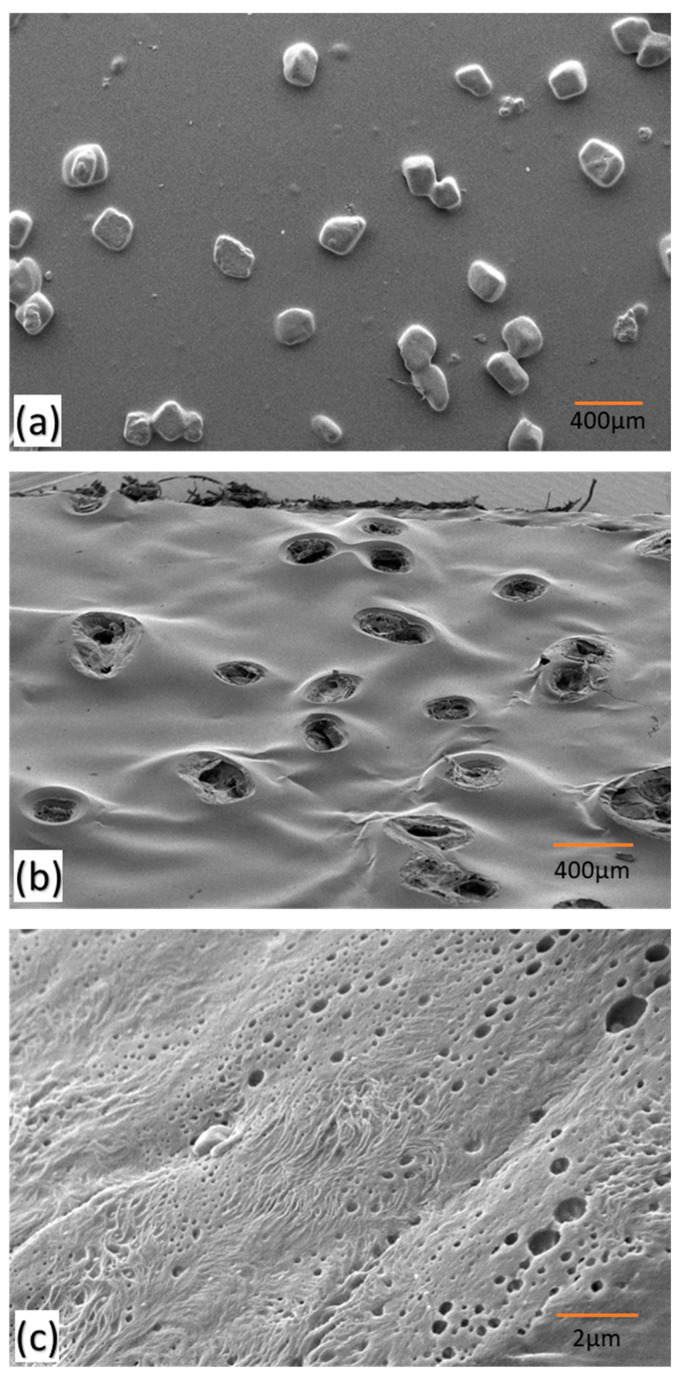
SEM images of LDPE sorbent (**a**) with cavities in it—top view, (**b**) with cavities in it—bottom view, and (**c**) a closer view of the surface showing micropores.

The SEM of the LDPE films revealed the structure of the cavities, where filler was covered from the top by LDPE, resulting in cavities with openings from the bottom (see Figure 4a). The superficial covering occurs due to the high density of the filler, which leads to its settling during spin-casting. Consequently, the filler was exposed to the atmosphere from the bottom side, and once it was removed from the thin film via dissolution in water, hollow cavities were formed. These cavities played a significant role in oil sorption due to the increase in surface area. These cavities range between 150–200 µm, corresponding to the size of the filler used. The number density of cavities, based on the amount of filler used, falls in the range of 400–600 per cm^2^. A closer view of the surface of the polymer thin-film sorbent is seen in Figure 4b, revealing the presence of micropores ranging from 500 nm to 1 µm on the surface due to polymer-solvent phase separation followed by annealing. These micropores facilitate oil penetration and retention in the cavities due to capillary action, adhesive, and cohesive forces, resulting in high uptake and retention capacities [36].

**Figure 5:**
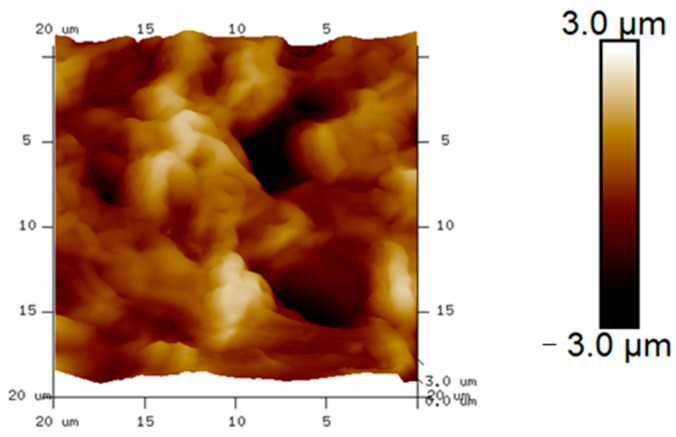
Surface roughness of the thin film using AFM.

It has been reported in the literature that oil sorption and oil retention increase with higher roughness of the sorbent surface [45]. To analyze the roughness and surface morphology of the swellable thin films, the AFM image of the thin films was examined, as shown in Figure 5. The average surface roughness on the thin film was observed to be 840 nm. These results demonstrated that the LDPE thin films possess sufficient roughness to provide high oil intake and retention properties. The crests and troughs in the image represent the micropores present in the thin films.

**Figure 6:**
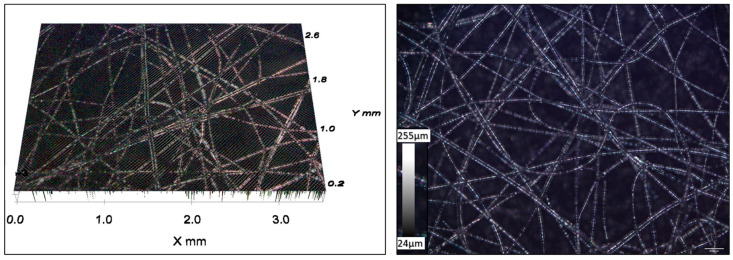
Optical microscopy images of nonwoven PP fibrous sheet.

Figure 6 displays the optical microscopic image of the nonwoven sheet, revealing a structural arrangement characterized by a microfiber diameter of around 50 μm. The presence of these nonwoven fibers is important in offering structural support, enabling rapid oil penetration and ensuring effective separation between the swellable films [46]. By placing this fibrous sheet between the swellable thin films, it serves as a facilitator, allowing oil to permeate easily and, consequently, reducing saturation time.

### 3.1. Oil Separation Studies

**Figure 7:**
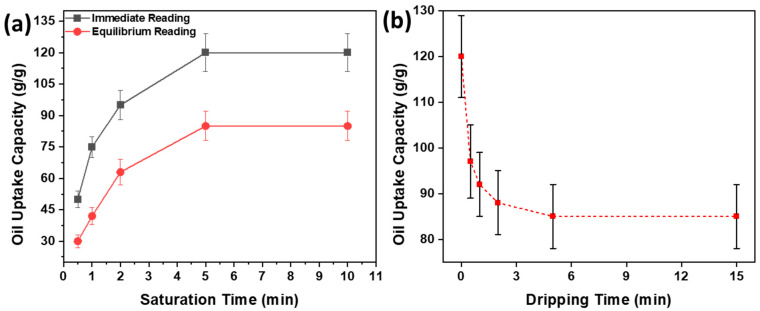
Effect of (**a**) saturation kinetics (**b**) dripping kinetics on oil uptake capacities.

The effects of saturation and dripping times on oil uptake capacity are shown in Figure 7. It can be observed in Figure 7a that immediate and equilibrium oil uptake capacities are very low for a saturation time of 0.5 min, i.e., 50 *g*/*g* and 30 *g*/*g*, respectively. However, as the saturation time increases, the immediate and equilibrium oil uptake capacities improve and reach the maximum uptake capacities in 5 min. This increment is because the longer the contact of the sorbent with the oil, the more sorption will occur until it reaches the equilibrium. Figure 7b shows the dripping time required to reach equilibrium oil uptake capacity is 5 min. This represents the amount of oil dripped from the sorbent, which is loosely connected to the sorbent, and ceded to gravitational forces. Thus, with a lapse in time, some amount of oil is drained and some is retained by the sorbent due to adhesive and cohesive forces.

**Figure 8:**
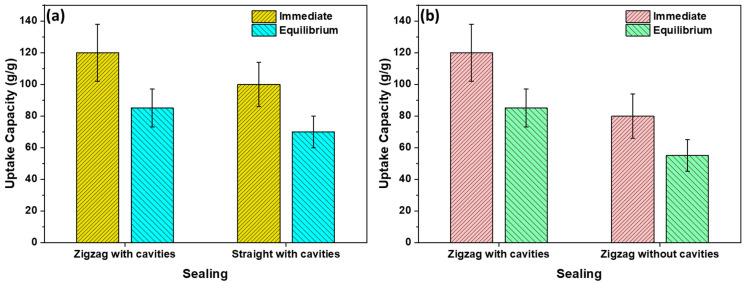
A comparison of oil uptake capacities for (**a**) zigzag-shaped sealing vs. straight-shaped sealing and (**b**) zigzag-shaped sealing with and without cavities.

Figure 8 demonstrates a comparison of oil uptake capacities when sealed in different shapes i.e., zigzag-shaped or straight-shaped. Additionally, to understand the presence and absence of cavities. Because of its higher retention capacity, the zigzag-shaped sealing with cavities exhibits a significantly greater oil uptake capacity when compared to sorbents lacking both cavities and zigzag-shaped sealing.

### 3.2. Oil/Water Selectivity 

**Figure 9:**
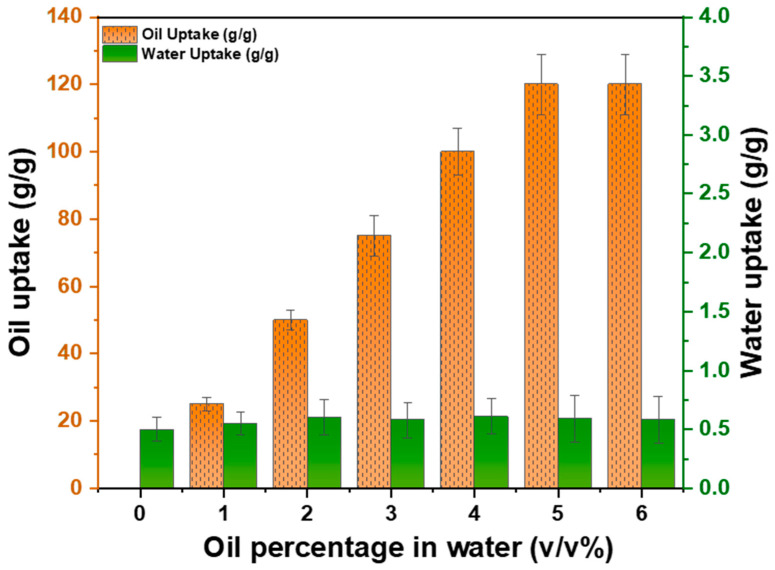
Oil–water selectivity of the sorbent in various oil–water suspensions.

To assess the oil–water selectivity of the sorbent, various percentages of oil in water were investigated. It was observed that the stacked sorbent absorbed the maximum amount of oil from oil–water suspensions until it reached the equilibrium oil uptake capacity of 120 *g*/*g*. Furthermore, the water uptake capacity did not exceed 0.63 *g*/*g* for oil/water suspensions under observation. This demonstrates that the sorbent has low water retention and high selectivity for oil. It was observed that the higher the oil content in the oil–water emulsion, the greater the oil sorption, reaching the maximum uptake capacity, see Figure 9.

### 3.3. Contact Angle

**Figure 10:**
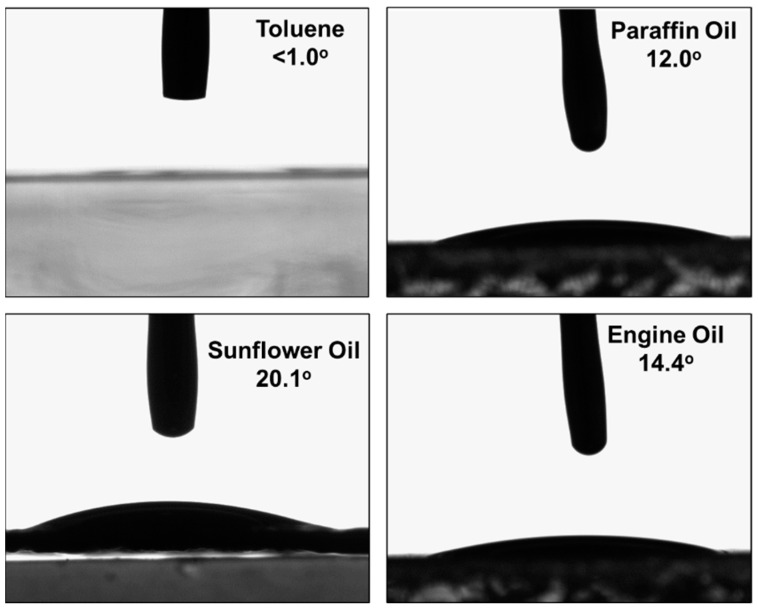
The contact angle of different oil sorbates.

Oleophilicity represents the capability of the material to attract oil [47].The surface of the sorbent is oleophilic if the oil contact angle is less than 90° [48]. The oil contact angle of the swellable thin film is shown in Figure 10, demonstrating that the sorbent exhibits oleophilic behavior toward various oils. The oil contact angle is very low, ranging between 0° and 20°, indicating a high degree of oleophilicity. The order of the oil contact angle is observed to be toluene < paraffin oil < engine oil < sunflower oil, corresponding to the degree of oleophilicities shown by the sorbates.

### 3.4. Uptake Capacities for Different Oil Sorbates

**Figure 11:**
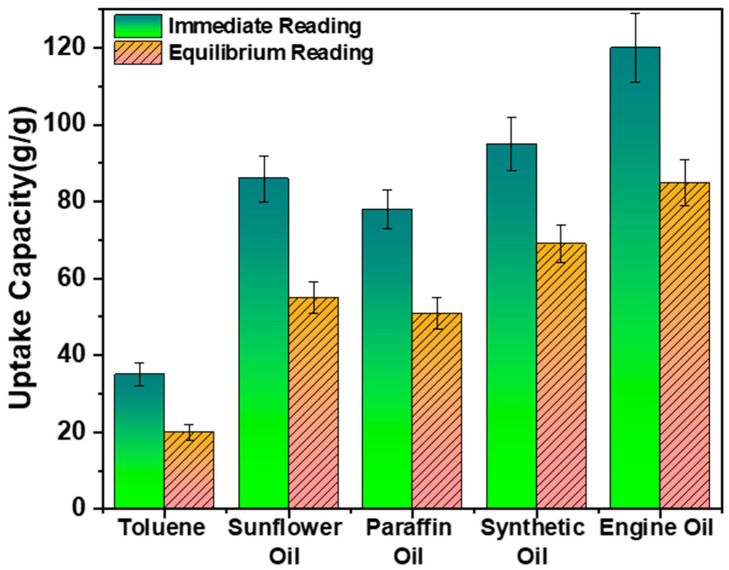
Comparison of different oil sorbates.

Various types of oils, including toluene, paraffin oil, sunflower oil, synthetic oil, and engine oil were used to examine as-prepared sorbent. It was observed in Figure 11 that the prepared sorbent showed high immediate and equilibrium oil sorption capacities for the selected oils, with toluene (35 *g*/*g* and 20 *g*/*g*) at the lowest and engine oil (120 *g*/*g* and 85 *g*/*g*) at the highest capacities. This shows these sorbents are suitable for organic solvents as well as viscous oils. The high uptake capacity is due to the combination of surface adhesion [49], capillary action due to the porous structure, cavities, and wire curvatures through the zigzag-shaped seal.

### 3.5. Comparison to Commercially Available Oil Sorbents

The stacked oil sorbent prepared in this study was compared to commercially available oil sorbents including 3M HP-255, Chemtex BP9W, and Corksorb based on immediate and equilibrium oil sorption capacities for engine oil. The results in Table 1 demonstrate the merit of the prepared sorbent as it exhibits high immediate and equilibrium oil sorption capacities for engine oil, i.e., 120 *g*/*g* and 85 *g*/*g*, respectively. The oil sorption capacities of 3M HP-255 were found to be 21 *g*/*g* and 18 *g*/*g* respectively. The as-prepared sorbent is effective for thin oil layers on water bodies. 

**Figure 12:**
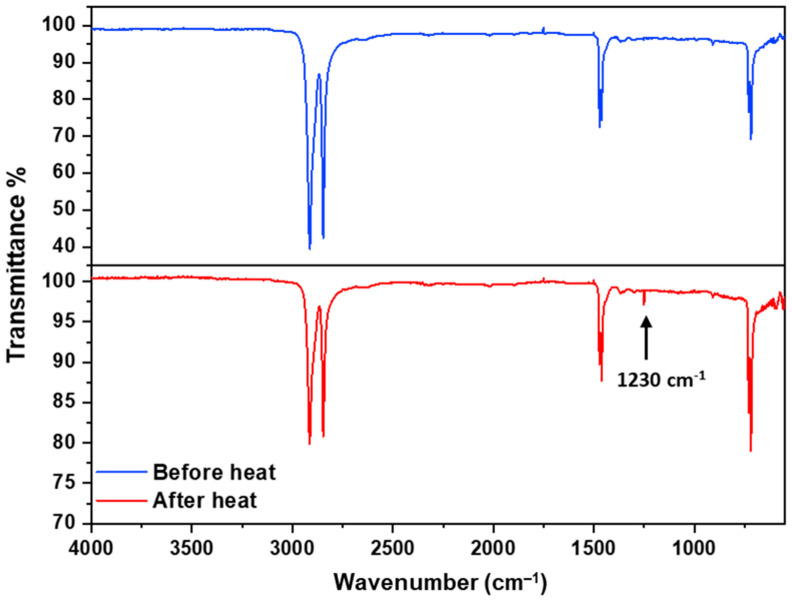
FTIR spectra of swellable thin films before and after heat treatment.

FTIR spectrum was used to study the bonding and molecular interactions of the polymer chains. Figure 12 shows the Fourier-transform infrared spectra of LDPE swellable thin film sorbent before and after annealing. The spectra of the film before heat treatment revealed the existence of C-H symmetric and asymmetric peaks at 2846 cm^−1^ and 2914 cm^−1^, respectively. The C-H bending peak appeared at 1462 cm^−1^, and the C-H rocking peak was found at 729 cm^−1^. Similarly, the film after heat treatment had the C-H symmetric and asymmetric peaks at 2847 cm^−1^ and 2915 cm^−1^, respectively; the C-H bending peak at 1464 cm^−1^; and an intense C-H rocking peak at 728 cm^−1^. The primary difference in the spectra before and after heating lies in the intensities of the percentage transmittance. The post-heat sorbents exhibitted more transmittance compared to the pre-heat sorbents. This indicates enhanced crystallinity in the after-heat sorbent, attributed to the close packing of polymer chains that reduces the gap between the chains, leading to a decrease in light-scattering and increased transmission. The lower transmittance in the pre-heat treatment suggests a more amorphous character. Additionally, an extra peak at 1230 cm^−1^ was observed in the after-heat-treated film corresponding to the C-O-C stretching peak. This suggests that upon annealing, the polymer chains crosslink through ether linkages, resulting in a stronger thin film due to enhanced intermolecular interactions, especially dispersion forces.

### 3.6. Effect of Physical Parameters

#### 3.6.1. Effect of Sorbent Preparation Conditions on Cavity Density of the Sorbent

**Figure 13:**
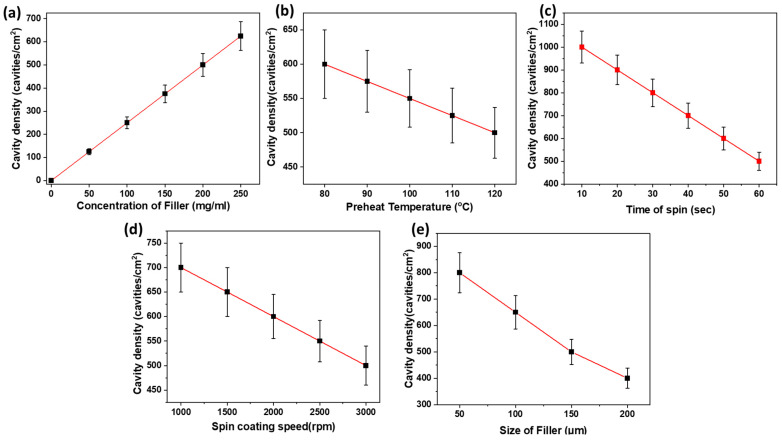
Effect of varying conditions on cavity density of thin films, (**a**) concentration of the filler vs. cavity density at speed of 2800 rpm, (**b**) preheat temperature vs. cavity density at speed of 2800 rpm and 130 mg/mL polymer solution, (**c**) time of spin vs. cavity density at speed of 2800 rpm and 130 mg/mL polymer solution, (**d**) spin-coating speed vs. cavity density at 130 mg/mlpolymer solution, and (**e**) size of the filler vs. cavity density at speed of 2800 rpm and 130 mg/mL polymer solution.

A thorough investigation was carried out to comprehend the effects of spin-casting speed, spin time, heating time, preheat temperature, size of the filler, and concentration of the filler on cavity density, cavity opening, strength, and thickness of thin films. The initial investigation focused on factors determining cavity density in thin films, which are presented in Figure 13, namely, speed, concentration, preheat temperature, size, and time of spin-casting. 

As depicted in Figure 13a, the density of cavities demonstrated a direct increase with a rise in filler concentration while maintaining a constant filler size of 150 μm. This direct proportionality arises from the fact that as the filler concentration increases, there is a greater retention of filler particles on the substrate, consequently leading to the formation of more cavities. 

For a constant concentration of 200 mg/mL and a filler size of 150 μm, an inverse relationship was observed for cavity density concerning preheat temperature, spin time, and spin-casting speed, as shown in Figure 13b–d. An increase in the preheat temperature of the substrate results in a longer duration of the polymer in a liquid state due to the slow transfer of heat from the solution to the substrate and then to the atmosphere, causing more filler to be expelled from the surface. Conversely, a fast transfer of heat at lower temperatures leads to the solidification of the polymer, retaining more filler. Simultaneously, an increase in spinning time extends the effects of centrifugal force, resulting in greater expulsion of filler, and consequently, fewer cavities formed. Similarly, an enhancement in the speed of rotation increases centrifugal forces, expelling more filler from the substrate, thereby decreasing the formation of cavities and cavity densities.

Likewise, at a given concentration (e.g., 200 mg/mL), a decrease in the size of the filler increases cavity density, as depicted in Figure 13e. This occurs because a reduction in filler size results in an increased number of particles for a given weight, leading to a rise in cavities and cavity densities.

#### 3.6.2. Effect of Changing Conditions on Cavity Opening of the Sorbent

**Figure 14:**
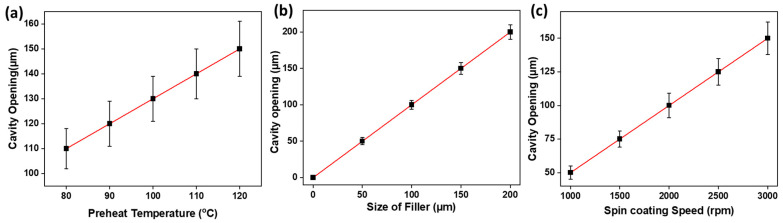
Effect of varying conditions on cavity opening of thin films, (**a**) preheat temperature vs. cavity opening at speed of 2800 rpm and 130 mg/mL polymer solution, (**b**) size of the filler vs. cavity opening at speed of 2800 rpm and 130 mg/mL polymer solution, and (**c**) spin-coating speed vs. cavity opening at 130 mg/mlpolymer solution.

Subsequently, we investigated factors influencing cavity opening, namely preheat temperature, filler size, and spin-casting speed. Maintaining a constant filler concentration of 200 mg/mL, cavity opening demonstrated a direct increase with preheat temperature, filler size, and spin-casting speed, as illustrated in Figure 14.

By varying the preheat temperature from 80 °C to 120 °C, we observed an escalation in cavity opening from 110 μm to 150 μm. This direct proportionality arises because the temperature increase prolongs the polymer’s liquid state, facilitating the settling of the filler to the bottom. Consequently, there is an augmented contact surface of the filler with the substrate, resulting in widened cavity openings.

Likewise, an increase in the filler size contributes to a larger cavity opening. The larger the filler size, the greater the contact surface, leading to an expansion in cavity opening. Concurrently, maintaining a concentration of 200 mg/mL, a size of 150 µm, and a preheat temperature of 120 °C, altering the spin-casting speed from 1000 rpm to 3000 rpm resulted in a threefold increase in cavity openings, as depicted in Figure 14c. This is attributed to the accelerated slithering of the polymer below the filler at higher speeds compared to lower speeds, leading to wider openings.

#### 3.6.3. Effect of Filler’s Concentration on Tensile Strength of the Sorbent

**Figure 15:**
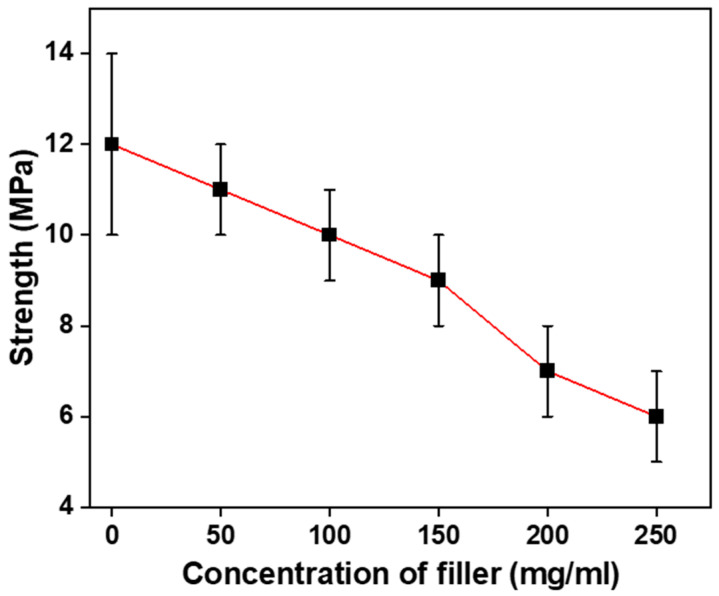
Effect of concentration of filler on strength of thin films at annealing temperature of 105 °C.

Furthermore, the impact of the concentration of filler on the strength of the thin film was studied as shown in Figure 15. As demonstrated in the figure, the tensile strength of the film decreases with the increase in the concentration of the filler. Increasing the concentration of the filler from 0 to 250 mg/mL reduces the strength of the swellable thin film. This is because the higher the filler concentration, the more the breakpoints and thus a decrease in strength. For the same range of the filler’s concentration, tensile strength has been decreased from 12 MPa to 6 MPa. 

#### 3.6.4. Effect of Spin-Casting Speed and Preheat Temperature on Film Thickness

**Figure 16:**
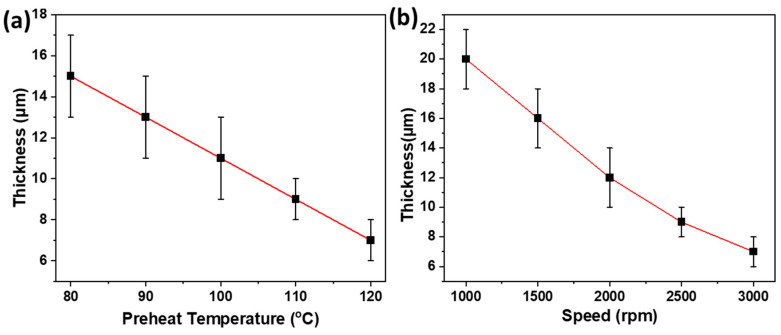
Effect of (**a**) preheat temperature and (**b**) spin-casting speed on film thickness saturation and dripping kinetics.

Film thickness was examined at various preheat temperatures and spin-casting speeds, revealing a decrease with an increase in both parameters, as depicted in Figure 16. The preheat temperature denotes the substrate’s temperature before polymer application for spin-coating. The elevation of the preheat temperature from 80 °C to 120 °C correlates with a reduction in film thickness from 15 µm to 7 µm, as depicted in Figure 16a. This inverse relationship is attributed to the prolonged liquid state of the polymer at higher preheat temperatures, leading to increased polymer expulsion and subsequent thinning of the films.

Similarly, an increase in spin-casting speed also results in a decrease in film thickness due to the heightened centrifugal force expelling more polymer from the substrate, as illustrated in Figure 16b.

## 4. Conclusions

The study successfully addressed the critical issue of plastic waste pollution by valorizing low-density polyethylene (LDPE) waste into 3D oleophilic sorbent films. The design involves the creation of swellable thin films, superposing them between a customized nonwoven polypropylene (PP) fabric and sealing them in a zigzag pattern. 

The developed sorbent demonstrated impressive oil uptake capacities, with immediate and equilibrium values of 120 *g*/*g* and 85 *g*/*g*, respectively, surpassing those of commercially available oil sorbents. Furthermore, it exhibited low water retention and a high selectivity for oil, making it a sustainable and valuable solution for selectively removing oil from water surfaces. The ability to address the issue of plastic waste pollution while providing a high degree of reusability is a significant contribution to environmental sustainability.

Through detailed characterizations, we gained insight into the unique features of the sorbent, including the presence of cavities that facilitate oil storage and retention, a surface with micropores aiding oil penetration and retention, and enhanced crystallinity due to annealing. These features contribute to the sorbent’s high oil uptake and retention properties.

The zigzag-shaped sealing technique, with its specific parameters and geometry, significantly improved the sorbent’s performance by enhancing pollutant retention and sorption capacity. The selection of LDPE waste as the primary material for the sorbent proved to be a practical choice, opening up opportunities for repurposing plastic waste for oil sorption applications.

This research not only presents an innovative approach to mitigating plastic waste pollution but also highlights the commercial potential in the field of oil sorption using repurposed materials. The results showcased the versatility of the developed sorbent by efficiently absorbing various oil types, thus extending its application to diverse scenarios.

## Figures and Tables

**Table 1:** Comparison to commercially available oil sorbents.

Type of Uptake Capacity	Sorbent (*g*/*g*)	3M HP-255 (*g*/*g*)	Chemtex BP9W (*g*/*g*)	Corksorb (*g*/*g*)
Immediate uptake capacity	120 ± 9	21 ± 4	17 ± 3	14 ± 2
Equilibrium uptake capacity	85 ± 6	18 ± 3	14 ± 2	11 ± 2

## Data Availability

The data presented in this study are available on request from the corresponding author.
